# Respite Care in Adult Day Services: An Updated Systematic Review From 2015 to 2025

**DOI:** 10.1093/geroni/igaf122.851

**Published:** 2025-12-31

**Authors:** Yawen Li

**Affiliations:** California State University, San Bernardino, San Bernardino, California, United States

## Abstract

Adult day services provide structured activities and supervised care for individuals with developmental disabilities and chronic health conditions such as diabetes and dementia, offering respite for caregivers. Building upon previous systematic reviews, this review synthesizes global evidence from 2015 to 2025 on the impact of ADS on caregiver burden, well-being, economic implications, utilization barriers, and service quality. A systematic search of PubMed, Cochrane Library, Google Scholar, and other databases identified over 25 relevant studies, including randomized controlled trials, observational studies and qualitative research. Findings indicate that regular ADS use is associated with reduced caregiver stress, improved mood, and physiological stress reduction. On ADS days, caregivers report lower negative affect and higher positive affect, with potential protective effects on stress biomarkers. ADS participation is linked to delayed nursing home placement, translating into cost savings. However, utilization remains suboptimal due to financial constraints, limited awareness, caregiver reluctance, and service availability. Facilitators include caregiver education, culturally tailored programming, financial assistance, and high-quality staff engagement. While early Cochrane reviews found inconclusive effects due to limited randomized trial data, recent observational and longitudinal studies provide stronger evidence of ADS benefits. Cost-effectiveness analyses support ADS as a strategy to delay institutionalization and reduce health care expenses. To maximize benefits, efforts should focus on increasing awareness, affordability, and cultural accessibility, alongside continued research to enhance service quality and utilization. Expanding high-quality ADS programs, such as ADS Plus, and addressing financial and logistical barriers are essential for optimizing respite care effectiveness and supporting caregivers.

